# Enhancing system safety in critical architectures: Augmented hypothesis testing with early design knowledge

**DOI:** 10.1371/journal.pone.0299633

**Published:** 2024-04-18

**Authors:** Fryad Khalid M. Rashid

**Affiliations:** 1 Technical College of Engineering, Sulaimani Polytechnic University, Sulaimani, Kurdistan Region, Iraq; 2 Computer Engineering Department, Komar University of Science and Technology, Sulaimani, Kurdistan Region, Iraq; Najran University College of Computer Science and Information Systems, SAUDI ARABIA

## Abstract

Hypothesis testing is a valuable method used to investigate ideas and test predictions arising from theories based on available data. In the context of critical system architecture, there is a need to effectively utilize hypothesis testing to identify faulty paths and improve system safety. This research aims to propose guidelines and best practices for presenting hypothesis testing in critical system architecture. The problem addressed in this study is the underutilization of hypothesis testing in life-critical system methods, resulting in a lack of identification of faulty paths. To address this challenge, we propose an enhanced pathway analysis technique that integrates error-derived information from a system’s architectural description, thereby augmenting traditional hypothesis testing methods. By investigating various paths, we aim to identify false positive and false negative errors in life-critical system architecture. Furthermore, the proposed method is validated based on specific validation criteria for each step such as system boundary, assumption, content/architecture, and traceability validations. Also, the method is evaluated based on our claims. The results of our research highlight the significance of tracing errors in early system knowledge. By leveraging the augmented hypothesis testing method, we are able to identify hazards, safety constraints, and specific causes of unsafe actions more effectively. The findings emphasize the importance of integrating early design knowledge into hypothesis testing for enhanced hazard identification and improved system safety.

## Introduction

Hypothesis testing, a rigorous statistical method, assesses claims about populations. It involves formulating two hypotheses: the null hypothesis (default assumption) and the alternative hypothesis (specific assertion). The goal, through data analysis, is to determine if there is enough evidence to reject the null hypothesis in favor of the alternative [1].

Hypothesis testing in safety-critical domains uses statistical analysis to assess hypotheses about system safety, reliability, and performance. It ensures adherence to standards, minimizing the risk of failures with severe consequences. This process involves formulating hypotheses on aspects like fault tolerance and response time, collecting and analyzing data to inform decisions on system safety and effectiveness [2, 3].

The main concern is understanding and managing errors in hypothesis testing for life-critical systems. Hypothesis testing involves decision-making using data, with a critical line as the threshold for accepting or rejecting the null hypothesis. Uncertainty about the true hypothesis and alternative hypothesis distribution properties can lead to two errors: Type I (False Positive) and Type II (False Negative).

Type I error happens when a positive result is obtained incorrectly, like alerting the driver in an adaptive cruise control system when the minimum safe distance is not breached. Conversely, Type II error occurs when a negative result is obtained incorrectly, such as failing to alert the driver when the minimum safe distance is breached. The critical value, serving as the significance level or threshold, guides decision-making for stakeholders or the system.

The problem statement raises key questions about Type I and Type II errors in life-critical systems: What are their consequences? Can they lead to incorrect system decisions, posing risks to lives and safety? Addressing these concerns requires exploring the criticality of these errors in life-critical systems.

- **RQ1:** How Type I and Type II errors could occur in the architecture of life-critical systems?
- **RQ2:** What strategies can be employed to control and mitigate Type I and Type II errors before the system makes critical decisions?

So,the aims of this study are to:

1. Investigate a practical approach that enables the identification and control of errors in life-critical system architecture, ultimately enhancing system reliability, safety, and decision-making processes.
2. Build a relationship between interdisciplinary research domains, especially data science and system safety.
3. Explore the specific challenges and considerations involved in applying hypothesis testing methods to life-critical systems.

In the literature review [4–13] have been observed that many data scientists and practitioners primarily focus on minimizing the occurrence of type I and type II errors by increasing the sample size of the data. However, despite these efforts, errors persist and remain unresolved. From a data science standpoint, such solutions are typically available only after the product has been built. Consequently, manufacturing companies often incur substantial costs to rectify these errors and reproduce the product, resulting in a significant expenditure of time and resources.

According the best knowledge of the author, from a system architecture perspective, a solution can be provided prior to the construction of the product. By identifying and addressing type I and type II errors during the early design phase, it becomes possible to control them before the system makes critical decisions. This approach offers several advantages, including cost and time savings, as errors can be detected and resolved before they escalate into larger issues during the manufacturing process.

Our research aims to provide valuable contributions in addressing the problem of errors in hypothesis testing in life-critical system architecture, and to answer the research questions:

1. We offer comprehensive guidance and effective strategies to empower stakeholders in identifying and rectifying faulty paths within the critical system architecture.
2. We develop a robust architecture for real-world safety-critical applications, including example such as adaptive cruise control system, to elucidate the phenomenon of error propagation across components.
3. We thoroughly discuss our findings and effectively showcase the capabilities of the proposed approach. To validate the method, we rigorously assess it against specific criteria and evaluate it in relation to our claims.

The motivation to address the problem of understanding and managing errors associated with hypothesis testing in life-critical system architecture stems from the following reasons:

- Enhancing Human Safety: Errors in decision-making can have severe consequences for human lives. Addressing Type I and Type II errors improves the accuracy of system decisions, minimizing risks and ensuring safety.
- Enhancing System Safety: Life-critical systems operate in high-stakes environments. Improving hypothesis testing enhances decision-making accuracy, leading to better safety measures and reduced risks.
- Risk Mitigation: Life-critical systems face complex environments with inherent risks. Addressing errors proactively manages risks, minimizing false positive and false negative outcomes that could lead to failures or inadequate responses.
- Addressing Effects of Errors: False positives and false negatives in hypothesis testing can have severe consequences. Comprehensive understanding allows the development of strategies to mitigate their occurrence and minimize their impact on system reliability and safety.

Addressing hypothesis testing errors improves human and system safety, mitigates risks, and minimizes the consequences of false positives/negatives in life-critical systems, aiming for highly reliable and safe systems that effectively respond to critical situations and improve well-being.

## Foundation

In this section, we provide the necessary background concerning hypothesis testing and life-critical systems to understand the concept of our work:

### 0.1 Understanding hypothesis testing

Hypothesis testing is a fundamental concept in the realm of statistics, serving as a structured and systematic approach to assess the validity of claims or hypotheses about populations or phenomena. Rooted in the scientific method, hypothesis testing provides researchers with a rigorous framework to draw meaningful conclusions from data while considering the uncertainties inherent in real-world observations. At its core, hypothesis testing involves the formulation of two competing hypotheses: the null hypothesis (H0) and the alternative hypothesis (H1). The null hypothesis represents the default or no-effect assumption, while the alternative hypothesis posits a specific assertion to be tested. Researchers collect data and perform statistical analyses to determine whether the evidence supports rejecting the null hypothesis in favor of the alternative hypothesis [14].

For instance, consider a scenario in medical research where a new drug is being evaluated for its effectiveness in treating a certain condition. The null hypothesis might assert that the new drug has no significant effect, while the alternative hypothesis suggests that it does. By conducting a clinical trial and analyzing the results, researchers can assess whether the observed improvements in patient outcomes are statistically significant enough to reject the null hypothesis and support the effectiveness of the new drug [15].

In [16],presents a structured framework comprising four essential steps for the application of the hypothesis testing method within a specific scenario. These steps encompass:

1. Formulate Hypotheses: Begin by explicitly stating the null hypothesis (H0) and the alternative hypothesis (H1). The null hypothesis represents the status quo or no effect, while the alternative hypothesis posits a specific effect or change.
2. Establish Decision Criteria: Define the criteria that will guide the decision-making process. This typically involves setting a significance level (alpha), which determines the threshold for accepting or rejecting the null hypothesis based on the strength of evidence from the sample data.
3. Compute Test Statistic: Calculate the appropriate test statistic based on the collected sample data. The choice of test statistic depends on the nature of the data and the hypotheses being tested. The test statistic quantifies the degree of deviation from the null hypothesis.
4. Make Informed Decision: Utilize the test statistic in conjunction with the established decision criteria to make an informed decision about the null hypothesis. When the test statistic aligns with the critical region established by the significance level, your course of action will involve either rejecting the null hypothesis in support of the alternative hypothesis or abstaining from rejecting the null hypothesis.

Furthermore, the decision outcomes have been categorized into four distinct possibilities [17]:

1. True Negative (TN): This outcome occurs when the null hypothesis (H0) is accurate, and the test correctly fails to reject it.
2. True Positive (TP): In this case, the alternative hypothesis (H1) is true, and the test correctly leads to the rejection of the null hypothesis (H0).
3. False Positive (FP/Type I Error): This situation arises when the null hypothesis (H0) is actually true, but the test incorrectly leads to its rejection. It represents the probability of mistakenly rejecting a true null hypothesis.
4. False Negative (FN/Type II Error): Here, the alternative hypothesis (H1) is true, yet the test fails to reject the null hypothesis (H0). This outcome signifies the probability of erroneously retaining a false null hypothesis.

By understanding these steps and outcome categories, researchers can conduct hypothesis testing more effectively and interpret the results accurately within a given context. In essence, hypothesis testing provides a structured methodology to bridge the gap between data and conclusions while accounting for the uncertainty that arises from various sources. It empowers researchers to make informed decisions, draw meaningful insights, and contribute to the advancement of knowledge across a multitude of fields.

### 0.2 Understanding life-critical systems

Within this section, we offer a comprehensive introduction to error ontologies—informative frameworks designed to assist users in contemplating potential errors within architecture fault modeling:

#### 0.2.1 Open Source Platforms

Both system safety experts and data scientists can harness the power of modeling languages to enhance the safety of the systems under their scrutiny or construction. In this context, we provide readers with insightful guidance to cultivate familiarity with key tools and concepts, namely the Architecture Analysis and Design Language (AADL) [18], Error Model Annex (EMV2) [19], and the Open Source Architectural Tool Environment (OSATE) [20], along with their practical implementation. AADL and OSATE have been selected for their notable attributes, including pre-existing support for our methodology through an array of plugins or extensions, and their established presence within the safety analysis community. By embracing these tools, stakeholders can effectively integrate our approach and make significant strides in bolstering system safety and analysis.

In order to establish a solid foundational comprehension of a pivotal aspect of our work, it becomes imperative to elucidate several key terminologies within the Architecture Analysis and Design Language (AADL) framework. These terms lay the groundwork for comprehending error behavior analysis as well as the captured hazards for a specific component [21]:

- Error Behavior: This pertains to the depiction of how distinct system components exhibit incorrect behavior, revealing deviations from their expected operational norms.
- States: These signify the various operational conditions that a component can undergo, encompassing both regular and abnormal states.
- Events: Errors manifest as events within the system, indicating anomalous occurrences that diverge from the anticipated behavior.
- Transitions: These illustrate the sequence of movements from one state to another, elucidating how error events instigate the progression of a component from a normal state to an irregular one.
- Hazard Properties: Hazards are stipulated within the component specification, categorized by their severity and priority levels. This classification facilitates the identification and prioritization of potential risks associated with the component.

By acquainting oneself with these fundamental AADL terms, readers can pave the way for a comprehensive understanding of our work, encompassing error behavior analysis, hazard visualization, and the pivotal role these terminologies play in illuminating intricate system dynamics.

### 0.3 Error ontology

The AADL Error Annex serves as a comprehensive guide detailing the methodology of representing diverse error types within a hierarchical framework. This systematic approach is instrumental in enhancing safety analysis efforts. Additionally, the annex introduces a pivotal notion—the concept of error classifications. This concept plays a crucial role in organizing and categorizing the myriad fault types that can potentially emerge during the analysis process.

The term “error type” functions as a categorical label, serving as a means to discern the propagation of errors from one component to another. This label also encompasses internal error instances within the component, delineates the behavior of the component in the presence of errors, and elucidates the trajectory of error transmission from input to output ports. In practice, the error label is employed as the foundation for condition declarations used in identification processes [22].

The Error Annex model further facilitates the portrayal of error behavior inherent to architectural components. This behavior is articulated using a state machine format, uniquely enabling the tracking of errors back to their origins. Beyond this, the model empowers analysts to discern the ripple effects of an error in one module on other interconnected components [23].

Error types serve as a valuable tool for stakeholders, offering a mechanism to elucidate potential component failures and establish connections between these failures and specific error events. For instance, consider a sensor malfunction: this can manifest as an erroneous reading (referred to as a value error), stemming from overheating. Alternatively, the sensor might skip a reading (termed an item omission) due to radiation interference, or it might altogether cease providing readings (recognized as a service omission) due to low power conditions [24].

The error ontology provides a comprehensive categorization of errors, neatly grouping them into six primary types, as detailed in [22, 24]:

- Service Errors: This category encompasses errors that transpire during the provision of a service. Within service errors, we identify omission errors—indicating the absence of any service delivery, and commission errors—highlighting instances where an unintended service was dispensed.
- Value Errors: Within this type, errors tied to service values are delineated. These encompass incorrect values, values that lie beyond the expected range, or stuck values—wherein the same value is repeatedly transmitted.
- Timing Errors: Errors linked to service scheduling are categorized here. This involves both early and late item delivery, as well as delayed service occurrences.
- Replication Errors: This category pertains to errors emerging within the delivery of replicated services. An example could be similar replicated programs being dispatched at distinct time intervals.
- Concurrency Errors: This category encompasses errors arising from concurrent system actions, such as the simultaneous execution of two tasks. Concurrency errors also encompass race conditions and mutual exclusion errors within memory management.
- Login Control Errors: This category involves errors associated with the implementation of access control systems. Within this context, authentication and authorization errors serve as illustrative examples.

By employing this systematic classification, the error ontology offers a structured approach to understanding and characterizing errors across different dimensions of service delivery and behavior.

### 0.4 Architecture fault modeling and analysis

This framework lends critical support to the automated assessment of quality attributes for dependable systems, including safety, reliability, and security. The integration of architecture fault modeling with the AADL error model annex empowers the augmentation of architecture models with insights into fault occurrence, malfunctioning, and fault propagation behaviors, effectively addressing dependability concerns within safety-critical systems [22]. This methodology operates across three levels of abstraction [21]:

- Component Behavior Analysis: At this level, safety analysis takes shape, encompassing probabilistic reliability and availability assessments. Furthermore, the status of a component during failure modes becomes discernible. For instance, the transition from operational to failure mode can be triggered by incoming error propagation or internal faults. Notably, components retain the capability to self-recover from failure.
- Compositional Abstraction Analysis: This tier amalgamates the diverse fault models of individual components into a unified global fault model for the entire system. Consequently, safety analysis can be conducted holistically for the entire system, foregoing the need to scrutinize each component in isolation.
- Error Propagation Analysis: This level delves into the effects of error propagation on the system’s operational landscape. Through the propagation of errors between components, the analysis illuminates how errors can impact the system’s functionality. As a result, this level becomes a pivotal tool for hazard identification and comprehensive error impact analysis.

Through the synergy of these levels, the architecture fault modeling and analysis methodology emerges as a robust framework for enhancing dependability and safety within complex systems.

## Related work

Within this section, we unveil our earlier endeavors pertaining to a critical architecture designed for contemporary medical devices, such as pacemakers. We also broaden our exploration of related works to encompass the ongoing research initiatives that strive to ensure the safety of life-critical systems. We conducted a comparative analysis with the previously described related works to assess and contextualize our own contributions.

In [4], our prior works, with the growing integration of Internet of Things (IoT) devices into daily life, their safety is paramount. This study focuses on ensuring IoT system safety through software development analysis. The method aims to analyze IoT safety during the design phase, addressing concerns stemming from the complexity of IoT interactions. Medical system analysis validates the method’s effectiveness in identifying hazards from software faults. Using standardized methods, the approach considers errors and hazards during design. The study shows that this approach identifies hazards in IoT systems without specialized knowledge. By introducing early hazard analysis in the Software Development Life Cycle, the approach identifies more hazards and safety constraints for successful critical IoT systems.

In [5], authors reveal critical transitions in various scientific domains where systems undergo abrupt shifts between states. Examples encompass epileptic seizures, climate changes, and ecosystem shifts. Early warning signals, a mathematical tool, have emerged to predict these transitions successfully across disciplines. Yet, not all transitions are detectable (false negatives), and signal appearance doesn’t invariably signify imminent change (false positives). Certain systems consistently exhibit early warning signals despite lacking critical transitions. This study aims to identify such systems, preventing misinterpretations. It also proposes strategies to counter systematic false positives and validates these insights using real-world data.

In [6], authors present cooperative vehicular communication networks to enhance traffic safety via safety applications. While technological aspects are extensively studied, uncertainty surrounds communication’s feasible safety support due to undefined requirements and overlooked false positive/negative reduction. This study investigates conditions for vehicular communication to aid safety applications. Authors identify safety application requirements, exemplified by Forward Collision Warning, prioritizing zero false positives/negatives. Authors determine the viable vehicle density under realistic communication conditions meeting requirements and quantify relaxation of ideal prerequisites for scalability and zero false rates.

In [7], as machine learning becomes more integrated into products, minimizing false negatives and false positives in binary classification gains importance. Existing bias methods are often ineffective. Author proposes a novel approach that reduces these errors without major performance changes. By adjusting input values post-pre-training, author achieves improved recall or precision without significant F1 score reduction across various data sets and models.

In [8], the researchers present strategies encompass two key components: (a) predicting impending failures and (b) implementing corresponding actions when a failure is anticipated. This study introduces a pioneering reliability model encompassing accurate and erroneous predictions, as well as both preventive measures and recovery preparations. The paper offers closed-form solutions for evaluating availability, reliability, and hazard rate.

In [9], Deep learning has excelled in image recognition, spurring interest in safety-critical applications like medical image interpretation and autonomous driving. As automation grows, addressing failure modes in deep learning becomes vital. This paper surveys self-monitoring techniques for machine-learning algorithms, focusing on uncertainty quantification. Authors apply these methods to semantic segmentation, which categorizes images semantically. Authors explore false positive and false negative errors, presenting new detection techniques.

In [10], this study introduces a validation framework for the System Theoretic Process Analysis (STPA) hazard analysis technique, addressing its growing industry relevance and lack of validation approaches. The framework focuses on assessing comprehensiveness, accuracy, and credibility through theory-based guiding questions. Using a formative approach, stakeholders can systematically improve the analysis. Drawing from disciplines like risk science and system dynamics, the framework’s practical utility requires validation through testing.

The referenced doctoral dissertation [11] emphasizes the demand for novel safety analysis approaches capable of addressing the intricacies inherent in IoT and cyber-physical systems. It also underlines the crucial need for the development of inventive resiliency methodologies, ensuring continuous functionality despite failures. Importantly, the author introduces the notion of utilizing Artificial Intelligence (AI) as a potential solution to enhance the overall safety and reliability of these intricate systems.

Another doctoral dissertation [12] emphasized the significance of traceability as a foundational requirement for establishing valid safety requirements. The researcher outlined three pivotal components essential for verifying safety constraints: hazard identification, hazard analysis, and software safety requirement tracing.

The author [13] highlights that while security concerns dominate discussions around the Internet of Things (IoT), the safety of devices is often sidelined, despite its critical importance. IoT devices are complex conglomerates of interconnected systems, making them susceptible to security breaches and device malfunction. Ensuring device safety is paramount to prevent adverse impacts on their operational environment. For instance, a wearable medical device could harm a patient, or a software glitch in a vehicle could lead to an accident. Notable examples of potentially hazardous IoT devices encompass wearable medical devices, vehicles, thermostats, and various smart home devices.

## Method

**Fig 1:**
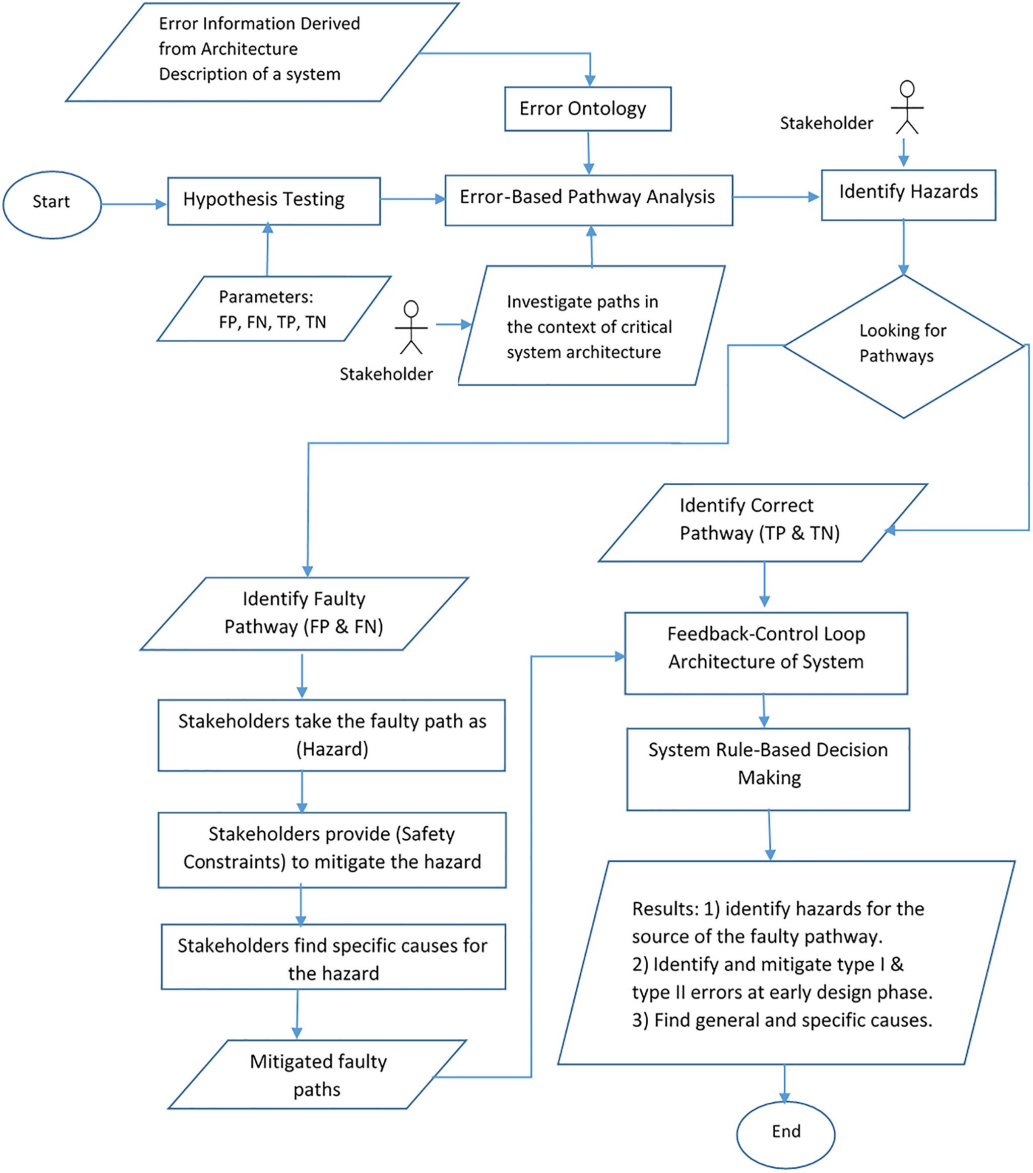


In this section, Error-Based Pathway Analysis Method has been established as a practical top-down safety analysis approach as shown in Fig 1:

As a reminder, the hypothesis testing involves the integration of two samples of normal distributions to create a contingency table, often referred to as a confusion matrix. This table consists of two dimensions: “actual” and “predicted.” Each row in the matrix represents an instance with a predicted value, while each column represents the corresponding actual value. The resulting matrix provides four important parameters: True Positive (TP), True Negative (TN), False Positive (FP), and False Negative (FN).

In addition, the error ontology is a hierarchical structure that categorizes different types of errors to assist in hazard analysis. It introduces the concept of error types to classify the errors that can propagate within a system. It defines an error event that occurs when a fault is activated and an error behavior state associated with each failure mode type.

The hypothesis testing method and the error ontology concept play crucial roles in life-critical system architecture by aiding in hazard identification and mitigation through error-based pathway analysis. Stakeholders within the architecture utilize these tools to identify various types of pathways, including the correct pathway (true positive/true negative) and the faulty pathway (false positive/false negative). By considering the faulty pathways as hazards, stakeholders can establish safety requirements to mitigate them effectively. Furthermore, stakeholders strive to pinpoint the specific causes behind these hazards. Consequently, it can be inferred that early detection and mitigation of false positives and false negatives during the design phase prevent the system from making incorrect decisions.

Following the principles of software architecture, each component comprises three crucial elements: input port, path, and output port. Stakeholders must proactively define potential errors related to a specific component, including the error source, error path, and error destination. For instance, if an incoming error (e.g., missing value or data) has been predefined for a component, the component itself handles it. However, if no predefined handling mechanism exists, the error becomes a new input for the component, traverses the component along its path, and reaches the output port. Subsequently, the error propagates through the communication links to other components, impacting the system’s overall interaction. Therefore, it is essential to isolate such errors before the system makes critical decisions.

To analyze the pathways and ensure system safety, cascading components are categorized into two formats: two-way and three-way communication formats:

- In the two-way communication format, two components are connected in a cascading manner to establish a communication setup. Any error produced by the first component propagates to the second component through the communication link, potentially causing the failure of the second component. These faulty paths also affect the system’s interaction and must be isolated before critical decisions are made.
- In the three-way communication format, three components are interconnected in a cascading manner, forming a communication link. Errors produced by the first component propagate through the second component and the communication links, ultimately reaching the third component. The third component may fail due to the error transmitted through the second component.

Fig 1 Error-Based Pathway Analysis Method, all in all, the incorporation of the hypothesis testing method and the error ontology concept into the life-critical system architecture enables stakeholders to identify and mitigate hazards, differentiate between correct and faulty pathways, establish safety requirements, and ensure that the system avoids making erroneous decisions.

To apply the error-based pathway analysis method to life-critical application examples, system analysts or architects should follow these key steps:

1. **Construct architecture:** During this pivotal step, system architects and analysts collaboratively develop a comprehensive feedback control loop system architecture for the identified life-critical system. This architecture encompasses the essential components of the system, including sensors, controllers, actuators, and the controlled process. The feedback control loop architecture provides analysts with a holistic view of the intricate interaction among these components, offering valuable insights into their collective functionality and operational synergy.
2. **Incorporate hypothesis testing parameters into the architecture:** This crucial step involves identifying and incorporating hypothesis testing parameters within the system architecture. Stakeholders are empowered to assess the parameters associated with false positives (FP), false negatives (FN), true positives (TP), and true negatives (TN) in the contingency table. The analysis of each parameter is guided by error ontology principles, enabling a thorough evaluation of the system’s performance and enhancing stakeholders’ understanding of its strengths and weaknesses.
3. **Identify the Correct Pathway (True Positive/True Negative):** In this step, stakeholders focus on identifying the pathways within the system where all elements, including the source, path, and destination, operate without any issues. This comprehensive evaluation encompasses both individual components and their interactions. The identified correct pathways are then integrated into the feedback control loop architecture, enabling the system to make informed decisions based on rule-based criteria.
4. **Identify Faulty Pathway (False Positive/False Negative):** This step empowers stakeholders to identify pathways associated with type I and type II errors through meticulous error propagation tracing. The error ontology framework serves as a valuable tool, enabling the identification of faulty pathways as hazards and the establishment of safety constraints to mitigate these risks. Additionally, stakeholders actively investigate the specific causes behind the identified hazards. To support stakeholders in this process, we propose two key strategies:
  **A) Deep understanding and tracing errors within individual components:** Stakeholders must possess a comprehensive understanding of system components and utilize effective tracing techniques to identify type I and type II errors occurring within individual components. By closely monitoring the occurrence and propagation of errors within a component, stakeholders can proactively mitigate internal failures.
  **B) Identification of communication pathways among components:** The communication pathways between cascading components play a critical role in life-critical systems. Stakeholders need to identify and analyze these communication pathways to trace the flow of errors from one component to another. This analysis enables stakeholders to gain insights into error propagation and take necessary measures to mitigate potential risks.
5. **Safety Architecture Design:** A comprehensive safety architecture is meticulously implemented by seamlessly blending the identified safety constraints into the overall system architecture. This integration ensures that safety aspects are robustly incorporated into every aspect of the system, promoting overall system reliability and risk mitigation.

## Results and analysis

In this section, we will analyze the results of the proposed method after applying it to various types of life-critical application scenarios:

**Scenario 1:** *What will happen if the sensor in the Adaptive Cruise Control System (ACCS) estimates incorrect values for the distance of the car in front of its own car (e.g., the sensor estimates the distance to be close enough, but in reality, it is not) during driving due to an internal failure?*

**Fig 2:**
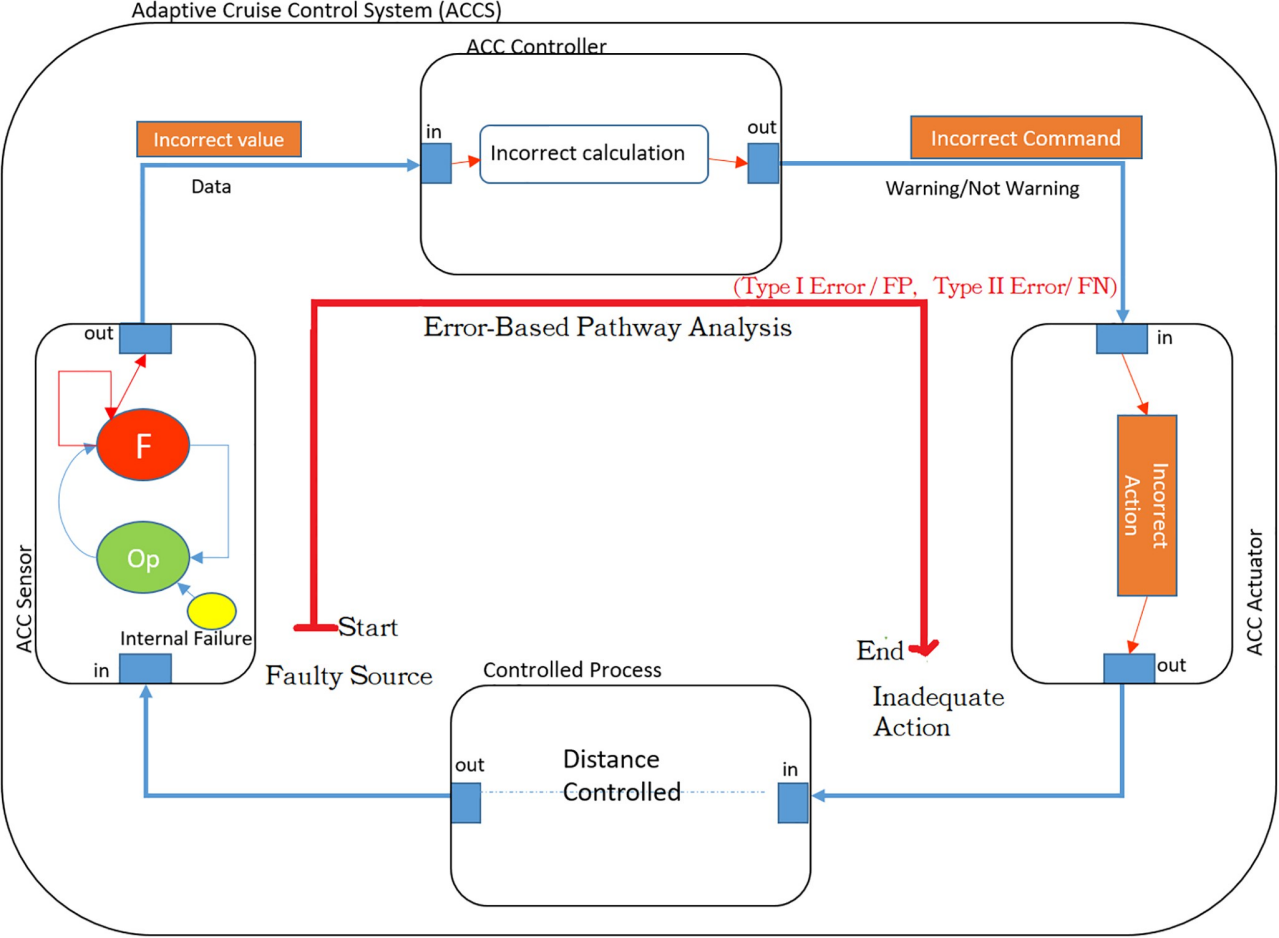


Fig 2 Feedback Control Loop Architecture, augmented with hypothesis testing and error propagation, the sensor initially starts in the operational state (OP). However, if the sensor detects an internal failure, it transitions to the failed state (F). As a result, the sensor becomes a potential source of error events. If the sensor successfully recovers from the internal failure, the error is contained within the sensor itself and does not affect the subsequent components. However, in cases where the sensor fails to recover, the error propagates through the outgoing port and follows an error propagation path towards the controller.

As shown in the Fig 2, due to an internal failure, the ACCS sensor generates inaccurate distance readings. These incorrect values propagate through the outgoing port and follow an error propagation path towards the controller. Within the controller, the process model begins calculating distances based on the received inaccurate values. Consequently, the controller is led to make incorrect decisions, potentially resulting in the transmission of inaccurate commands to the ACCS actuator. Consequently, the ACCS actuator performs inappropriate actions based on these flawed commands.

**2) The hypothesis testing parameters have been identified in the ACCS architecture**.

In Table 1, different parameters have been identified which have been related to the ACCS (Adaptive Cruise Control System) as a life-critical system, include:

True Positive (ACCS-TP*(1)): The ACCS controller correctly warns the driver when the minimum distance is presented. This indicates that the controller functions appropriately by issuing a warning based on the actual distance.
False Positive (ACCS-FP*(2))—Type I Error: The ACCS controller incorrectly warns the driver when the minimum distance is not presented. This means that the controller makes an erroneous decision, issuing a warning command even though the distance is not below the minimum threshold. This situation can lead to unnecessary driver distraction.
False Negative (ACCS-FN*(3))-Type II Error: The ACCS controller fails to warn the driver when the minimum distance is presented. This is a critical issue as the controller overlooks available data and fails to send a warning command when it should. Such an omission can increase the risk of a potential collision.
True Negative (ACCS-TN*(4)): The ACCS controller does not warn the driver when the minimum distance is not presented, which aligns with normal system operation. In this case, the controller appropriately abstains from issuing a warning as there is no available data indicating a need for it.

**Table 1:** Identify FP/FN/TP/TN in ACCS application example.

	Min. Distance presented	Min. Distance not presented
Positive Result (Warn)	ACCS-TP*(1)	ACCS-FP*(2)– Type I Error
Negative Result (Not Warn)	ACCS-FN*(3)–Type II Error	ACCS-TN*(4)

3) The two correct parameters, True Positive (ACCS-TP*(1)) and True Negative (ACCS-TN*(4)), provide valuable insights into the accuracy and reliability of the ACCS (Adaptive Cruise Control System). These parameters indicate that the ACCS is functioning correctly, as the ACCS sensor accurately reads the distance values and the ACCS controller performs accurate calculations based on the received sensor data. This accurate processing by the ACCS controller enables it to make correct decisions. Finally, the ACCS controller sends the appropriate commands to the ACCS actuator, ensuring that the actuator performs the correct action.

4) As shown in Fig 2, the remaining two faulty parameters in the pathway, False Positive (ACCS-FP*(2))—Type I Error and False Negative (ACCS-FN*(3))-Type II Error, indicate that the ACCS is experiencing issues and requires rectification as follows:

- The faulty path (i.e. red soled line) as shown in the Fig 2 has been identified based on the mentioned of the two key strategies which have been described in the step three of the methodology: So, the faulty path: [ACCS sensor (source)]—> [ACCS controller (path)]—> [ACCS actuator (destination)] has been described as follows: An internal failure within the ACCS sensor results in the transmission of incorrect values to the ACCS controller. As a consequence, the ACCS controller performs inaccurate calculations based on these received values. Consequently, an incorrect command is sent from the ACCS controller to the ACCS actuator. Finally, the ACCS actuator, acting upon this erroneous command, executes an incorrect action.
- To identify source of the faulty path, the error (incorrect estimation values) has been identified as hazard (H) as follows: The error ontology categorizes incorrect estimations as instances where the ACC system provides inaccurate values. In this case, the identified hazard is the presence of incorrect estimation values for the car originating from the ACC system.
- Safety Constraint (SC1) has been provided to mitigate the hazard (H) as follows: ACC system should provide correct values for the distance.
- To address the faulty parameters (False Positive (ACCS-FP*(2))—Type I Error and False Negative (ACCS-FN*(3))-Type II Error) within the path, safety constraints have been provided to mitigate it as follows:
  Safety Constraints (SC2): [False Positive (ACCS-FP*(2))-Type I Error]: The ACCS controller should warn the driver when the minimum distance is presented at any time.
  Safety Constraints (SC3):[False Negative (ACCS-FN*(3))-Type II Error]: The ACCS controller should not fail to warn the driver when the minimum distance is presented.
- For the end or destination of the pathway, the general and specific causes have been identified for the inadequate action of the ACCS actuator as follows: The General Cause behind the transmission of an incorrect command from the ACCS controller to the ACCS actuator can be attributed to an internal failure within the ACCS sensor. This failure disrupts the proper functioning of the sensor, leading to the generation of inaccurate values. Consequently, the Specific Cause for the ACCS actuator performing an incorrect action can be traced back to the propagation of these erroneous values as an error event from the ACCS sensor to the ACCS actuator. The actuator, relying on faulty information, executes an inappropriate action, resulting in undesired behavior.

**Fig 3:**
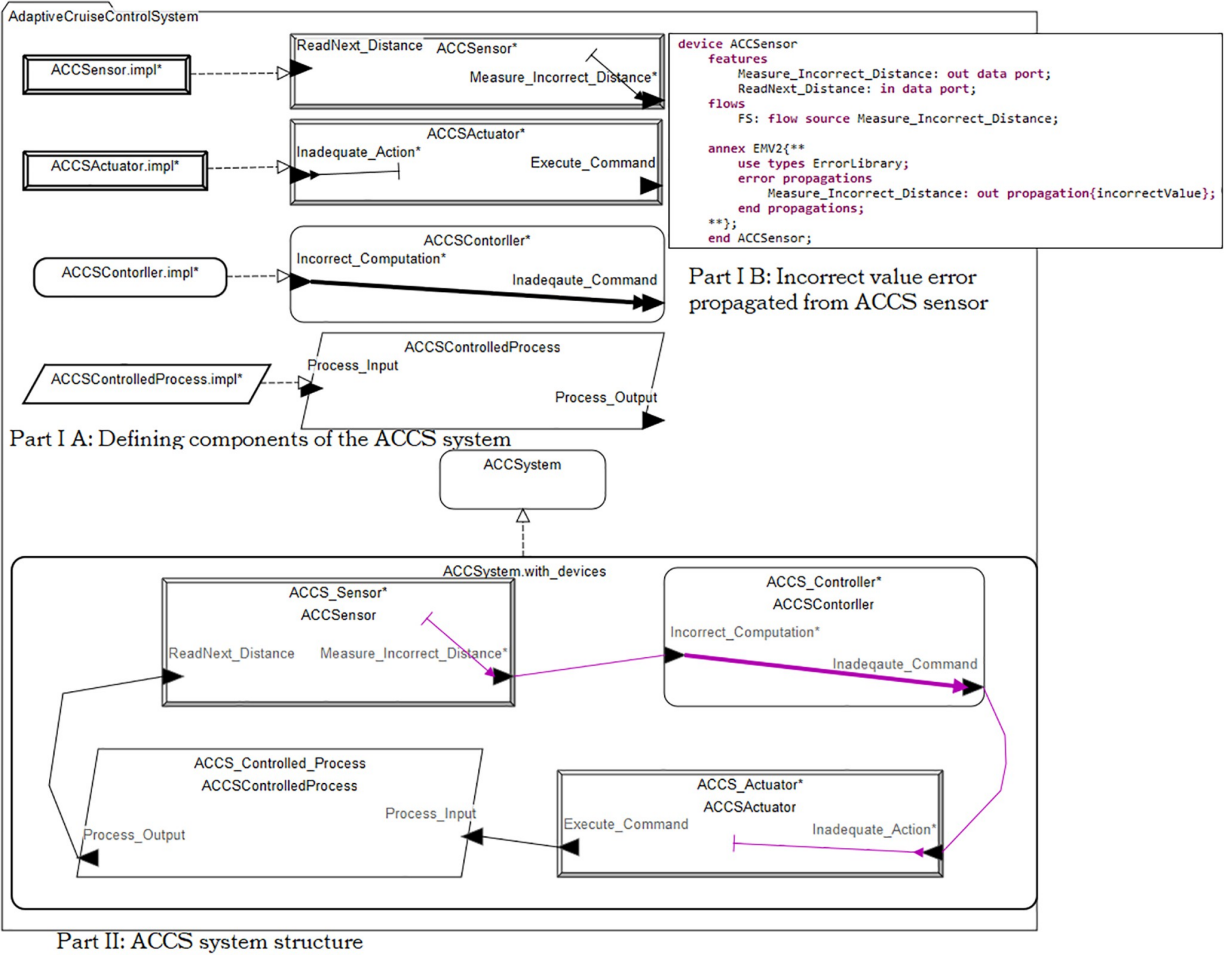


**5) The safety architecture construction as shown in Fig 3 represents the final crucial step in our methodology**. It involves integrating the outcomes of the preceding steps into the system’s architecture to address inadequate actions, identify potential hazards during the early stages of system design, establish safety constraints, and determine both general and specific causes of inadequate actions.

In this section, we focus on Scenario 1, which pertains to the ACC system’s architecture. To facilitate this analysis, we leverage the Architecture Analysis and Design Language (AADL) supported by the Open Source Architectural Tool Environment (OSATE).

Utilizing this framework, we develop a feedback control loop pattern and enhance it with critical error propagation information and hypothesis testing contributors. We meticulously document error events, propagated errors, states, and transitions in the error model (EMV2) for the major system components. This systematic approach ensures that potential safety issues are thoroughly addressed and mitigated during the design phase.

In order to comprehensively address and mitigate potential safety issues during the design phase, we employ a meticulous documentation approach that encompasses error events, propagated errors, states, and transitions within the error model (EMV2) for the major system components. By systematically documenting these elements, we ensure a thorough analysis of safety considerations, allowing for effective identification and resolution of potential hazards.

Fig 3 Error-Based Pathway Analysis Development for Scenario 1, based on the labels within it described as follows:

**(Part I A) Define ACCS components:** From the software engineering perspective, using AADL code we have defined the specification and implementation for each component of the adaptive cruise control system. For example, sensor specification has been defined as ACCSensor with input and output data ports and the sensor implementation has been defined as ACCSensor.impl. The definition of the specification help to see external view of the components and the definition of the implementation helps to see internal structure of the system.

**(Part I B) Error Propagation Analysis:** Incorrect value of the distance has been measured by the ACCS sensor due to the internal failure. As a result the error propagated throughout out event port and propagation path to the ACCS controller for processing.

**(Part II) ACCS system structure:** We described as follows:

1. When the ACCS sensor experiences an internal failure, it is recorded as an error event, causing the operational state of the sensor to change to a failed state. This failure impacts the ACCS sensor’s reading values, leading to incorrect values for the car in front (e.g., incorrect distance).
2. The internal failure results in the propagation of incorrect distance values throughout the event port and propagation path to the ACCS controller for processing as shown in Part I B.
3. The ACCS controller receives incorrect values from the incoming port and proceeds with computations. However, it fails to comprehend the propagated erroneous values, leading to the transformation of the propagated error into an error event, which shifts the controller’s state from normal to error. Consequently, the inaccurate computation results in the transmission of inadequate commands to the ACCS actuator, owing to the ACCS controller processing incorrect or abnormal data.
4. The inadequate command is transmitted to the ACCS actuator through both the control flow and the propagation path.
5. Upon receiving the incorrect command, the ACCS actuator behaves as if the car is too close to the vehicle in front, triggering a warning to the driver to apply the brakes. However, this warning is not based on the actual distance in reality. If the driver fails to respond, the system will automatically apply the brakes. Unfortunately, both scenarios may lead to an accident as the car performs an unsafe action based on incorrect values. In either case, braking suddenly in the middle of the road poses a risk of being rear-ended by the vehicle behind it.
6. The ACCS controlled process’s incoming port (i.e., process input) becomes the sink or destination of the error.

Summary of the results:

- A hazard (H) has been identified within the (source) of the faulty pathway. Also, safety constraint (SC1) has been provided to mitigate it.
- Safety constraints (SC2 and SC3) have been provided to mitigate the Type I and Type II errors within the (path) of the faulty pathway.
- General and specific causes have been provided for the (end/destination) of the faulty pathway.

## Method validation

In this section, we direct our attention to a set of critical validation criteria that have been introduced as a validation framework in the doctoral dissertation authored by [10]. These criteria are of significant importance in the validation of safety analysis methods. These encompass system boundary validation, which is crucial for systems with life-critical implications. Additionally, we delve into assumption validation, particularly pertinent to scenarios involving safety-critical considerations. Further, we scrutinize content and architecture validation, as well as traceability validation. Each of these facets serves as a yardstick to gauge the efficacy of our approach. The safety analysis method we put forth is tailored to confront the validation challenges posed by these diverse criteria. By integrating targeted validation inquiries within each relevant criterion, our proposed method comprehensively addresses the intricacies of validation.

This section is divided into four distinct sub-sections, each corresponding to a specific step within the error-based pathway analysis method.

**Fig 4:**
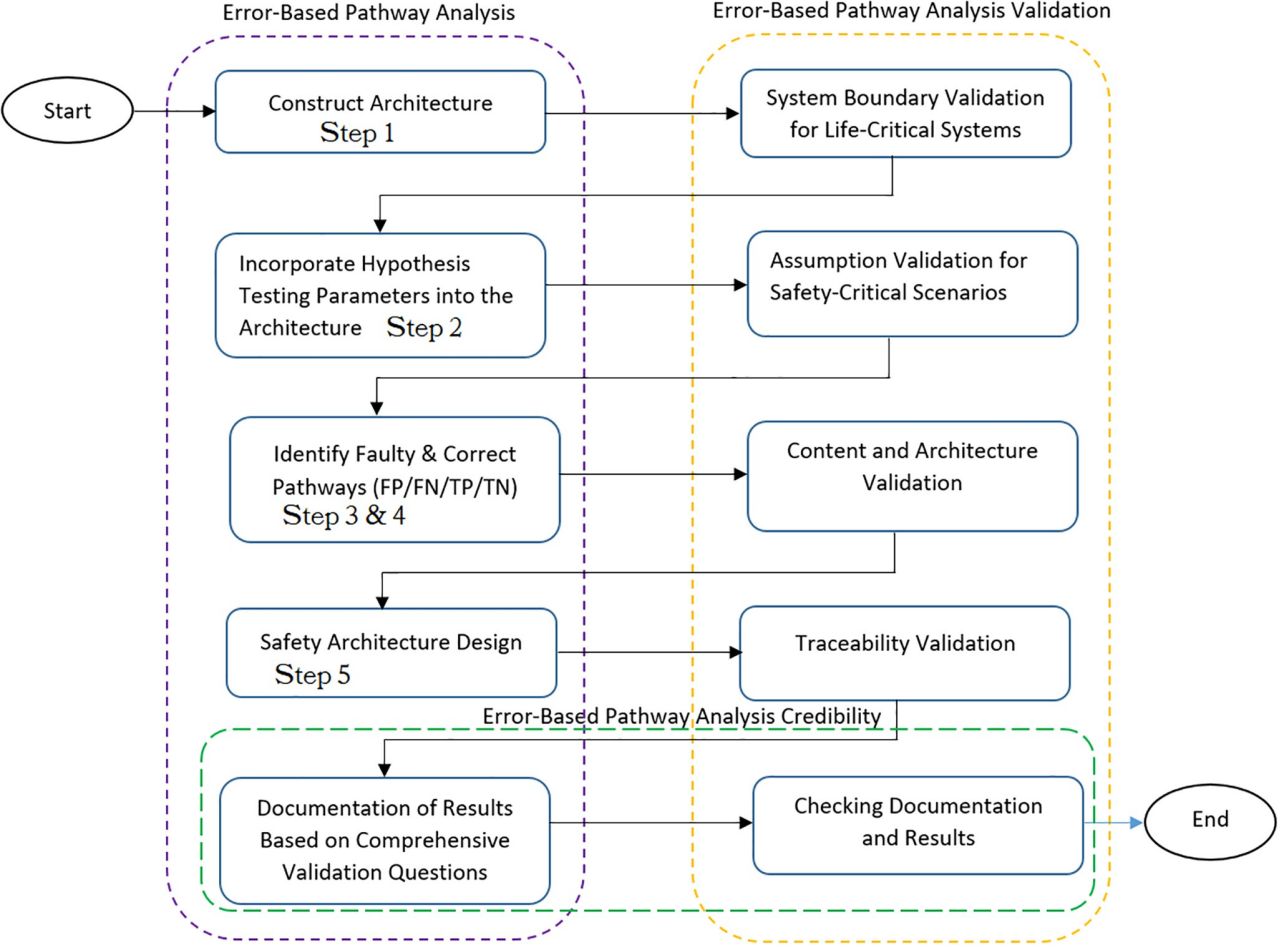


Fig 4 Validation Framework for the Error-Based Pathway Analysis Method, it offers a robust means to thoroughly validate each phase of the proposed safety analysis method, employing a set of comprehensive validation inquiries. These inquiries encompass every facet of the error-based pathway analysis method, delineating explicit validation criteria and accompanying guiding questions within the subsequent subsections. The guiding questions are thoughtfully crafted through a dual approach: firstly, they are culled from existing literature and thoughtfully adapted for seamless integration into the error-based pathway analysis method context. Secondly, they are formulated based on the author’s extensive knowledge and experience in the realm of safety analysis and its attendant validation standards. Moreover, the validation framework provides a set of high-level guiding questions, acknowledging the potential need for further refinement in practical scenarios or when applied within diverse life-critical domains.

So, to validate the step one of the error-based pathway analysis method, we focus on the following validation criteria to measure the validity it:

1. **System boundary validation for life-critical systems:** In system-level safety analysis, before identifying potential hazards and safety constraints, it is vital to define the system and its boundaries based on the analysis’s purpose and context. The system boundary distinguishes what components the system can control from those it cannot. The boundary should be tailored to align with the analysis’s objectives, allowing for flexibility, such as including or excluding human actions depending on the specific case. Validating the system boundary involves evaluating the effects of boundary changes. Stakeholders play a critical role in this process by assessing how altering the boundary would impact identified hazards and safety requirements. This ensures a comprehensive understanding of risks and supports the implementation of appropriate safety measures [25, 26].
  So, to validate the step one of the error-based pathway analysis method, we focus on the following validation criteria to measure the validity it:
  - *Is the system boundary explicitly defined for the proposed method?* This question aims to ensure that the system boundary is clearly and precisely defined for the method’s application. By explicitly defining the system boundary, stakeholders can better understand the scope of validation, particularly for safety-critical systems, enabling the identification of hazards at the system level and errors at the component level. Additionally, stakeholders can contribute safety constraints to mitigate potential hazards effectively.
  - *Does the system boundary align with the specific objectives of the analysis?* It is crucial to verify that the defined system boundary directly corresponds to the analysis’s intended objectives. A well-aligned system boundary ensures that the analysis remains focused on the intended scope and relevant context. In the case of life-critical systems, the architecture of the system should align with error propagation analysis, which originates from the description of system components’ architecture.
  - *Do the system stakeholders possess control over all identified components within the system boundary?* Validating the level of control that system stakeholders have over all components within the system boundary, such as sensors, controllers, actuators, and the controlled process, is essential. This validation process allows stakeholders to trace errors back to their source, identifying potentially faulty components and understanding how errors propagate from one component to another.
  Once the system boundary validation questions have been thoroughly addressed within the proposed method, it becomes possible to assess its effectiveness and suitability within the defined system boundary. A method that has undergone robust validation, with a clearly defined boundary that aligns with its purpose, ensures the generation of dependable results. This, in turn, aids in identifying potential hazards and enables the implementation of appropriate safety constraints, reinforcing the overall safety of the system.
2. **Assumption Validation for Safety-Critical Scenarios:** Assumptions play a significant role in safety-critical analyses, and their accurate assessment is vital for reliable results. The assumption validation process consists of two essential steps to ensure a comprehensive understanding of their impact on the analysis [27–30]: First step, relevance assessment, in this step, all identified assumptions are carefully examined to determine their relevance to the problem situation. Stakeholders evaluate whether the opposite of any specific assumption would significantly alter the results of the method. If changing an assumption would not have a substantial effect on the outcomes, it indicates that the assumption might not be highly relevant to the analysis. Second step, importance and certainty determination, the step involves assessing the degree of importance and certainty associated with each relevant assumption. This evaluation is based on stakeholders’ judgment about the perceived impact of the assumptions on the analysis. Understanding the significance and level of confidence in the assumptions allows for a more informed decision-making process.
  So, to validate the step two of the error-based pathway analysis method, we focus on the following validation criteria to measure the validity it:
  - *Are the assumptions (i.e. safety-critical scenarios) fully identified and accurately described for the proposed method?* The assumptions (i.e. safety-critical scenarios) have been fully identified in different safety-critical domains such as adaptive cruise control, medical devices and automated door control systems. Ensuring a comprehensive identification and clear description of all assumptions is crucial for a sound analysis foundation.
  - *If the opposite of any particular assumption were true, would it have a substantial effect on the identified hazards and safety constraints?* Absolutely. The evaluating the potential impact of changing specific assumptions helps to identify critical factors that significantly influence safety-critical scenarios.
  By rigorously answering these questions within the proposed safety analysis method, safety-critical analyses can attain a higher level of reliability and effectiveness, ultimately resulting in enhanced safety measures and more robust risk mitigation strategies. Also, assumption validation plays a crucial role in fostering agreement among stakeholders and deepening their understanding of the analysis’s underlying foundation. Thoroughly validating assumptions lends credibility to the analysis and ensures that safety-critical scenarios are more accurate and comprehensive, leading to better-informed decision-making and safer outcomes.
3. **Content and Architecture Validation:** According to [31], content validity of a model refers to its ability to accurately represent the variables and parameters found in the real system. It ensures that the components included in a risk model adequately reflect the knowledge about the system and its relevance in the real world [32]. In the context of error-based pathway analysis, content validity testing becomes essential for validating the components within the feedback control loop architecture.
  In [33] described content validation as a qualitative form of validation where analysts determine whether the necessary structural relationships, essential for fulfilling the analysis’s purpose, are incorporated. Stakeholder When applying content validity to an application example, the components and the relationships between them in their validation process need to be both examined.
  To ensure utmost clarity and precision in the proposed error-based pathway analysis method, the validation strives to rigorously assess the precision and thoroughness of both the included components in the system architecture and their functional relationships. As a result, this meticulous test effectively validates both the contents (identifying the components present in the model) and the architecture (analyzing the interconnections between components).
  So, to validate the step three and four of the error-based pathway analysis method, we focus on the following validation criteria to measure the validity it:
  - *Does the constructed architecture encompass all relevant system components?* Ensuring the model incorporates all pertinent components (i.e., sensors, controllers, actuators, and the controlled process) is crucial for achieving accuracy and effectiveness. The feedback control loop architecture connects these components, facilitating error tracing at the component level and hazard identification at the system level.
  - *Does the constructed architecture encompass all relevant functional relationships between system components?* Validating the presence of all essential relationships between components guarantees an accurate representation of the system’s dynamics. The system components are interconnected based on their component-structure, connection lines, and component functionality: *Component-structure*: The component-structure includes in/out ports, facilitating the exchange of data and different types of error events among components. *Connection lines*: These lines are utilized to represent both the nominal control flow and the paths for error propagation among components. *Sensor*: This component is responsible for measuring values within the system. *Controller*: The controller plays a crucial role in regulating system parameters and transmitting commands to actuators. *Actuator*: As per the controller’s instructions, the actuator executes commands within the system. *Controlled Process*: This component illustrates the internal processes within the controller and effectively implements its decisions.
  By thoroughly addressing these questions through the content and architecture validity test, the error-based pathway analysis method can be fortified, providing a more reliable understanding of the system’s behavior and enabling the implementation of more effective risk mitigation strategies.
4. **Traceability Validation:** Maintaining traceability between different error-based pathway analysis outputs is essential to facilitate system changes without the need to redo the entire analysis. Ensuring traceability becomes particularly crucial in safety-critical scenarios, where every potential risk must be traceable to one or more errors, and each error should be linked to one or more system-level hazards. To mitigate these hazards effectively, safety constraints must be established.
  It is important to note that traceability does not necessarily entail a one-to-one relationship. A single safety constraint might serve to prevent multiple hazards, and conversely, multiple safety constraints could be associated with a single hazard. By establishing such comprehensive traceability, the system’s safety can be continuously monitored and managed, allowing for targeted improvements and modifications while upholding a robust safety standard.
  So, to validate the step five of the error-based pathway analysis method, we focus on the following validation criteria to measure the validity it::
  - *Can the identified hazards be traced to relevant propagated errors?* The proposed safety analysis method ensures accurate traceability from hazards to propagated errors, allowing each safety-critical scenario to be connected to its underlying errors effectively. Stakeholders can confidently trace the relationship from the system-hazard level down to the component-error level and vice versa.
  - *Can the identified causes of failures be traced to all relevant hazards, propagated errors, and safety constraints?* The analysis’s comprehensiveness and reliability are strengthened by verifying that the causes leading to safety-critical scenarios can be traced back to their associated safety constraints, hazards, and propagated errors. The proposed safety analysis method allows stakeholders to trace the causes of errors (i.e., faults) and their resulting failures, as well as the causes of losses (i.e., hazards) and their mitigation through safety constraints, all based on the component and system levels.
  - *Are the traceability properly presented in the form of pathways?* The transparency and ease of understanding for stakeholders are enhanced by adequately documenting traceability in the form of clear and logically organized pathways. The pathway presentation facilitates tracing errors from the component level to the system level, guiding stakeholders through the faulty component, the error it would produce, and the destination of the error within the system.
  By addressing these key questions and ensuring robust traceability, the proposed safety analysis method proves its effectiveness in evaluating and mitigating safety-critical scenarios. Stakeholders can have confidence in making informed decisions and implementing necessary changes to maintain and enhance system safety.
5. **Checking Documentation and Results:** Ensuring the validity of each step in the proposed method is essential for bolstering the trustworthiness of our analysis. Credibility, in this context, denotes the level of confidence that stakeholders and decision-makers can place in the outcomes of our analysis. It encompasses the overall assessment of whether a risk evaluation can be relied upon and embraced. Credibility, however, is not solely contingent on the quality of the analysis but also extends to contextual factors that intertwine with the concept of trust within the realm of risk management. As a result, this section recommends the validation of documentation and the thorough examination of results as crucial steps to fortify the establishment of this credibility.
  Documentation checking is a critical process designed to guarantee accuracy, completeness, and up-to-date. It also underscores the importance of transparency and adequacy in documentation for the benefit of stakeholders, including those who may not be well-versed in the intricacies of the analysis technique. This validation step aligns harmoniously with the principle of quality assurance, which entails a thorough examination of the analytical process.
  During this step, the overarching process is meticulously scrutinized to ensure its overall coherence. To facilitate this assessment, stakeholders are tasked with evaluating the final documents and addressing the following inquiries:
  (a) Is the documentation of the error-based pathway analysis implementation sufficiently comprehensive and coherent?
  (b) Are the documentation formats intelligible to users and stakeholders who may lack familiarity with error-based pathway analysis?
  (c) Are the sources of uncertainty adequately and clearly documented?
  By addressing these questions, we aim to fortify the reliability and comprehensibility of our documentation, fostering an environment of trust and understanding among all stakeholders involved.
  In the realm of results checking, effective communication of the analysis and its validation findings plays a pivotal role in enhancing stakeholders’ comprehension of the entire analytical process. Typically, stakeholders are most concerned about whether their interests have been adequately taken into account throughout the analysis. Therefore, when presenting the results to stakeholders, it is crucial to clearly convey how and where their identified interests have been addressed within the analysis, with the ultimate goal of bolstering the analysis’s credibility.
  To facilitate this communication, consider addressing the following key questions when presenting results to stakeholders:
  (a) Has the hazard been identified as a source of the faulty pathway? If so, have safety constraints been provided to mitigate it?
  (b) Have Type I and Type II errors been identified within the path of the faulty pathway? If yes, have safety constraints been provided to mitigate them?
  (c) Have both the general and specific causes been thoroughly elucidated for the endpoint or destination of the faulty pathway?
  By addressing these questions, we can ensure that stakeholders gain a clear and comprehensive understanding of how their concerns and interests have been integrated into the analysis, thereby reinforcing the credibility of the entire process.

## Method evaluation

In this section, our attention is directed towards a comprehensive assessment of the top-down architectural safety analysis technique. Specifically, we will delve into the error-based pathway analysis method, which holds paramount importance in the development of architectures for life-critical systems. This method serves as a powerful tool for stakeholders to proactively mitigate the impact of errors by instituting safety constraints during the formulation of safety-critical architecture models.

Within our approach, we place a strong emphasis on the identification of safety constraints aimed at counteracting potential hazards originating from faulty pathways. We also concentrate on addressing type I and type II errors occurring in the middle of these pathways. Moreover, we are dedicated to uncovering both the overarching and specific causes underpinning unsafe actions situated at the culmination of these pathways.

To achieve these objectives, we seamlessly integrated an error ontology into the architecture model of an adaptive cruise control system, which is critical for ensuring safety in vehicular contexts. This integration paved the way for a meticulous examination of unsafe actions through a comprehensive error behavior analysis. Furthermore, it facilitated the identification of the precise factors contributing to these actions, achieved via an in-depth error state analysis.

In summary, our exploration involves a meticulous evaluation of the error-based pathway analysis technique within the context of life-critical systems. We present a method that encompasses the proactive establishment of safety constraints and the in-depth analysis of various error types and their underlying causes, all within the framework of a practical adaptive cruise control system architecture.

In this context, we subject our technique to a rigorous evaluation based on the following assertions:

### 0.5 Claim 1

The absence of an error-based pathway analysis method to effectively address the criticality of False Positive (FP) and False Negative (FN), or Type I and Type II errors, within complex cross-domain domains like data science and life-critical systems presents a significant challenge. This claim is substantiated by a tangible scenario wherein the adaptive cruise control system resulted in harm. Notably, this harm arose due to the inherent functionality of a safety-critical component, as exemplified in Section (scenario I).

#### 0.5.1 Evidence and explanation for Claim 1

- **Cross-Domain Challenges:** Complex cross-domain systems, especially those involving data science and life-critical applications, often exhibit intricate interactions and dependencies. The absence of a dedicated error-based pathway analysis method makes it challenging to assess the criticality of errors, specifically False Positives (FP) and False Negatives (FN), across these domains. The lack of such an approach leaves a gap in effectively addressing the nuanced challenges associated with different domains.
- **Tangible Scenario—Adaptive Cruise Control Harm:** The scenario presented in Section (scenario I) provides a concrete example of the consequences of not having an error-based pathway analysis method. In this case, harm resulted from the adaptive cruise control system, a life-critical application. The harm was directly linked to the inherent functionality of a safety-critical component, emphasizing the need for a systematic analysis method that considers error pathways and their criticality in such systems.

### 0.6 Claim 2

Our method, in conjunction with its complementary components, not only enhances the validation of prevailing hazard analysis approaches, such as those outlined in references [34–36], but also provides a multi-layered evaluation paradigm. Through the identification of safety constraints, hazard recognition, detection of False Positive (FP) and False Negative (FN) or Type I and Type II errors within flawed pathways, formulation and development of safety architecture, and the application of error propagation analyses in the initial design phase, our approach offers a holistic methodology surpassing conventional hazard analyses. A testament to this can be observed in our safety analysis of the adaptive cruise control architecture, detailed in Section (methodology and scenario I).

#### 0.6.1 Evidence and Explanation for Claim 2

- **Enhancement of Prevailing Hazard Analysis Approaches:** The reference to established hazard analysis approaches [34–36], indicates the integration of existing methodologies. By combining these with our proposed method, the validation of hazard analyses is strengthened. The incorporation of complementary components ensures a comprehensive evaluation paradigm that goes beyond traditional hazard analysis frameworks.
- **Multi-Layered Evaluation Paradigm:** Our method introduces a multi-layered approach, encompassing various stages of system development. This includes the identification of safety constraints, hazard recognition, and the detection of False Positives (FP) and False Negatives (FN) or Type I and Type II errors within flawed pathways. Additionally, the formulation and development of safety architecture, coupled with error propagation analyses in the initial design phase, contribute to a holistic methodology.
- **Concrete Application—Adaptive Cruise Control Safety Analysis:** The application of our approach to the safety analysis of the adaptive cruise control architecture, detailed in Section (methodology and scenario I), serves as a concrete testament to its efficacy. The specific details of the analysis demonstrate how our method addresses the complexities inherent in life-critical systems and surpasses conventional hazard analyses.

### 0.7 Claim 3

A significant advantage of our proposed error-based pathway analysis method for life-critical systems is its capacity for pre-implementation execution, thereby enabling early identification and mitigation of potential hazards. This stands in contrast to many prevailing techniques that defer safety assessment until the system is partially implemented. In support of this, we showcase in Section (Construct Architecture) the application of our approach in assessing the safety of an adaptive cruise control architecture ahead of the implementation phase.

#### 0.7.1 Evidence and Explanation for Claim 3

- **Pre-Implementation Execution Advantage:** The claim highlights a key advantage of the proposed error-based pathway analysis method, specifically its ability to operate pre-implementation. Unlike many existing techniques that delay safety assessment until the system is partially implemented, our method enables early identification and mitigation of potential hazards. This proactive approach enhances overall system safety.
- **Contrast with Prevailing Techniques:** The claim positions our method in contrast to prevailing techniques that defer safety assessments. This contrast emphasizes the significance of early hazard identification and mitigation, especially in life-critical systems. It addresses a common limitation in current practices and underscores the innovation introduced by our approach.
- **Concrete Application—Adaptive Cruise Control Safety Assessment:** The application of our method in Section (Construct Architecture) to assess the safety of an adaptive cruise control architecture ahead of the implementation phase provides concrete evidence of its pre-implementation execution capability. The details of this application showcase how potential hazards can be identified and addressed at an early stage, demonstrating the practical implications of the claimed advantage.

Our method effectively tackles all three aforementioned claims while simultaneously diminishing the necessity for specialized domain expertise, achieved by harnessing established safety analysis tools from diverse domains. By integrating hypothesis testing from the data science realm and leveraging error ontologies alongside error propagation analysis sourced from the AADL community, we seamlessly pinpoint safety requisites, architect sound safety frameworks, and scrutinize errors in the nascent phases.

Notably, this new approach obviates the need for deferring safety evaluation until the system attains partial realization, which is a common requirement in numerous methodologies. Instead, our methodology empowers early-stage hazard detection and mitigation. Moreover, the utilization of AADL, an open-source tool, adds to the merit of our approach. This tool is readily accessible and is fortified by a vibrant and expanding community, ensuring continuous support and enhancement.

In essence, our method harmonizes claims, diminishes the demand for specialized knowledge, and leverages established tools, thereby expediting safety analysis and augmenting the efficiency of safety evaluation in complex domains.

## Discussion

In this section, we thoroughly examine the capabilities of our proposed approach through a comparative analysis with related works. Additionally, we delve into the challenges encountered during the validation and evaluation of our method:

- In [4], departing from the methodology outlined in the referenced study, our approach is centered on the exploration of overlooked safety constraints through error-based pathway analysis. More precisely, we meticulously examine safety constraints designed to address errors transmitted from sensors to actuators within the feedback control loop architecture.
- In [5], deviating from the methodology outlined in the referenced study, we adopt a different approach to address the research objectives. So, our research is centered on heightening awareness regarding the safety of critical systems, aiming to mitigate the repercussions of both false positives and false negatives. Given the pervasive integration of sensors, controllers, actuators, and digitally enhanced objects equipped with computational and communication capabilities, life-critical systems are progressively permeating every facet of our daily existence. This pervasive integration underscores the urgency for heightened consciousness and in-depth safety assessments within the architecture of life-critical systems.
- In [6], diverging from the approach of the referenced study, our methodology centers on uncovering overlooked safety constraints through error-based pathway analysis. Specifically, we scrutinize safety constraints aimed at rectifying errors propagated from sensors to actuators within the feedback control loop architecture.
- In [7], diverging from the approach of this study, our methodology revolves around error mitigation during the initial design phase of the product. Notably, our method obviates the necessity for distinct experts to address these errors.
- In [8], our approach distinguishes itself by adopting a unique framework that traces errors and comprehends their propagation across components through a three-way communication format. Moreover, we employ a pre-established set of generic error categories, drawn from cross-domain analyses, rather than devising our own categories.
- In [9], our approach stands out for its emphasis on mitigating false positives and false negatives during the initial product design phase. By adopting our methodology, we aim to diminish the reliance on experts in diverse fields like data science and image recognition. In contrast, our method strives to decrease expert dependence, allowing safety analysis to occur earlier in the software development life cycle.
- In [10], our methodology provides a pathway to establish this validation framework, enabling a more comprehensive validation of safety requirements. Our approach involves diverse validation criteria tailored to each step of the safety analysis method we propose. This encompasses system boundary validation for life-critical systems, assumption validation for safety-critical scenarios, content and architecture validation, and traceability validation.
- In [11], our approach employs a distinct methodology where we construct a comprehensive framework for life-critical system architecture encompassing IoT and cyber-physical systems. The objective is to uncover any potential safety limitations that might have been missed during the initial design phase.
- In [12], our methodology, we provide a mechanism to achieve this traceability as a validation technique, serving to assess the validity of a key step within the proposed safety analysis approach. This enhances the thorough validation of safety requisites. Moreover, our work delves into various validation approaches, encompassing system boundary validation for life-critical systems, content and architecture validation, as well as assumption validation.
- In [13], While our methodology adeptly detects and addresses potential hazards and minimizes false positives/negatives during the early design phase, it’s essential to note that our proposed approach does not specifically cater to security concerns.

### 0.8 Method’s benefits

The error-based pathway analysis method, as described through its key steps, provides several notable benefits to support the claims:

1. **Comprehensive System Understanding:** The process of constructing the architecture enables system architects and analysts to develop a holistic view of the life-critical system. This comprehensive understanding extends to the intricate interactions among essential components such as sensors, controllers, actuators, and the controlled process.
2. **Enhanced Performance Evaluation:** Incorporating hypothesis testing parameters into the architecture facilitates a thorough evaluation of the system’s performance. Stakeholders can assess false positives, false negatives, true positives, and true negatives, gaining insights into the system’s strengths and weaknesses based on error ontology principles.
3. **Informed Decision-Making:**Identifying correct pathways allows stakeholders to focus on areas where all system elements operate without issues. Integration of these pathways into the feedback control loop architecture empowers the system to make informed decisions based on established rule-based criteria, contributing to effective and reliable operation.
4. **Proactive Hazard Mitigation:** The method empowers stakeholders to identify and mitigate hazards associated with faulty pathways, including type I and type II errors. Meticulous error propagation tracing, guided by the error ontology framework, facilitates the establishment of safety constraints and proactive measures to minimize the impact of potential risks.
5. **Root Cause Analysis and Internal Failure Mitigation:** Two proposed strategies, namely deep understanding and tracing errors within individual components, and identification of communication pathways among components, support stakeholders in conducting a thorough root cause analysis. This, in turn, enables the proactive mitigation of internal failures within the system.
6. **Communication Pathway Analysis:** By identifying and analyzing communication pathways among cascading components, stakeholders gain insights into the flow of errors from one component to another. This analysis is crucial in understanding error propagation, allowing stakeholders to take necessary measures to mitigate potential risks and enhance the robustness of the entire system.
7. **Robust Safety Architecture:** The method culminates in the meticulous implementation of a comprehensive safety architecture. Safety constraints identified during the analysis are seamlessly blended into the overall system architecture, ensuring that safety aspects are robustly incorporated into every aspect of the system. This, in turn, promotes overall system reliability and effective risk mitigation in life-critical scenarios.

### 0.9 Challenges in method validation

Here are challenges that need to be addressed to ensure the effectiveness and applicability of the proposed validation method for identifying error pathways, hazards, type I and type II errors, and safety constraints in a life-critical system architecture:

- Complexity Management: Life-critical system architectures can be highly complex, with numerous components and interactions. Managing the complexity while validating the method’s ability to identify error pathways and hazards without oversimplifying the system is a challenge.
- Data Availability and Reliability: Acquiring accurate and reliable data for validation can be challenging, especially for rare events or edge cases. Real-life data may not fully cover all potential error scenarios, making it difficult to comprehensively validate the method’s performance.
- Dynamic System Changes: Life-critical systems are subject to modifications and updates over time. The validation method must account for the dynamic nature of these systems and ensure that it remains effective even as the system evolves.
- Unintended Consequences: The validation process should consider the potential for unintended consequences when identifying and mitigating error pathways. An overemphasis on one error pathway or hazard could inadvertently introduce new risks elsewhere in the system.

### 0.10 Challenges in method evaluation

Evaluating the top-down architectural error-based pathway analysis technique for developing life-critical systems comes with several challenges. Here are the challenges associated with evaluating the claims and the overall approach:

- Substantiating Harm in Tangible Scenarios: Demonstrating harm resulting from the absence of error-based pathway analysis in life-critical systems may be ethically and practically challenging. Constructing scenarios that clearly show the impact of missing error analysis requires careful planning.
- Quantitative Assessment of False Positives and Negatives: Quantifying the criticality of False Positive (FP) and False Negative (FN) errors in complex domains like life-critical systems is difficult. Creating a robust quantitative measure that accounts for both the impact and consequences of these errors is a challenge.
- Standardization of Metrics: Establishing standardized metrics for evaluating the validation enhancement and multi-layered evaluation paradigm’s effectiveness can be challenging due to varying criteria used in different methodologies.
- Scenario Complexity and Diversity: Developing scenarios that comprehensively cover the complexity and diversity of life-critical systems while also integrating cross-domain elements from data science presents a significant challenge. Ensuring these scenarios accurately reflect real-world situations is crucial for meaningful evaluation.

Addressing these challenges in the evaluation process is essential for substantiating the claims, ensuring the proposed method’s practicality, and demonstrating its effectiveness in improving safety analysis and evaluation in complex and critical domains.

## Conclusion

In conclusion, integrating early design knowledge with hypothesis testing holds great promise for identifying a broader range of hazards and pinpointing specific triggers for unsafe actions within life-critical system architecture. Adhering to outlined guidelines and best practices enables practitioners to enhance safety measures and reduce risks in these systems.

The proposed safety analysis method not only reveals new hazards but also highlights the necessary safety constraints to mitigate these risks. This comprehensive framework empowers stakeholders to understand how errors can arise from individual components, spread among various elements, and potentially endanger system users.

The research emphasizes that tracing errors is a key strategy for uncovering previously unnoticed hazards in complex life-critical systems. The presence of errors doesn’t always lead to hazards; outcomes depend on causal factors and the specific system. The method operates on the premise that studying errors in life-critical domains generates a list of potential hazards relevant to other critical systems.

The validation of the proposed method for identifying error pathways, hazards, and safety constraints in life-critical system architectures presents significant challenges. These include managing complexity, acquiring reliable data, accommodating dynamic system changes, and considering unintended consequences. Addressing these challenges is crucial to ensure the effectiveness and applicability of the validation method. Also, evaluating the top-down error-based pathway analysis technique for life-critical systems presents challenges in substantiating harm, quantifying false positives and negatives, standardizing metrics, and incorporating scenario complexity. Addressing these challenges is crucial for a comprehensive evaluation of the approach.

Ultimately, this approach equips safety experts and data engineers with a powerful tool to systematically discover inherent hazards in device interactions, bolstering life-critical systems with effective countermeasures. To validate the approach, it will be implemented and rigorously compared with existing methods, followed by thorough testing on widely used systems to showcase its effectiveness in safety analysis.

## 1 Appendix: Illustrative scenarios

This appendix presents two carefully constructed scenarios that showcase the versatility of the proposed method in addressing a variety of life-critical applications:

**Scenario 2: Infant Incubator Air Temperature Anomaly** In this scenario, we explore the implications of an out-of-range air temperature reading within an infant incubator (isolette) due to an internal failure in the operator interface device (OID). The scenario delves into the potential consequences and responses that would be triggered by such an event.

**Scenario 3: Pacemaker Heart Sensing Anomaly** This scenario focuses on the situation where inaccuracies arise in the sensing values transmitted by the leads within the heart to the device controller monitor (DCM) in a pacemaker. Even though the patient’s heart rhythm may not actually necessitate pacing, erroneous signals trigger the pacemaker. The scenario examines the outcomes and actions that could ensue from this false stimulation.

Through these two distinct scenarios, the capability of the proposed method to address diverse life-critical situations is effectively demonstrated.

## Supporting information

S1 Fig (PNG)
