# Supplementary figures and images for: Enhancing system safety in critical architectures: Augmented hypothesis testing with early design knowledge
Source: PLoS One. 2024 Apr 18;19(4):e0299633. doi: 10.1371/journal.pone.0299633 (PMC11025889; doi:10.1371/journal.pone.0299633)

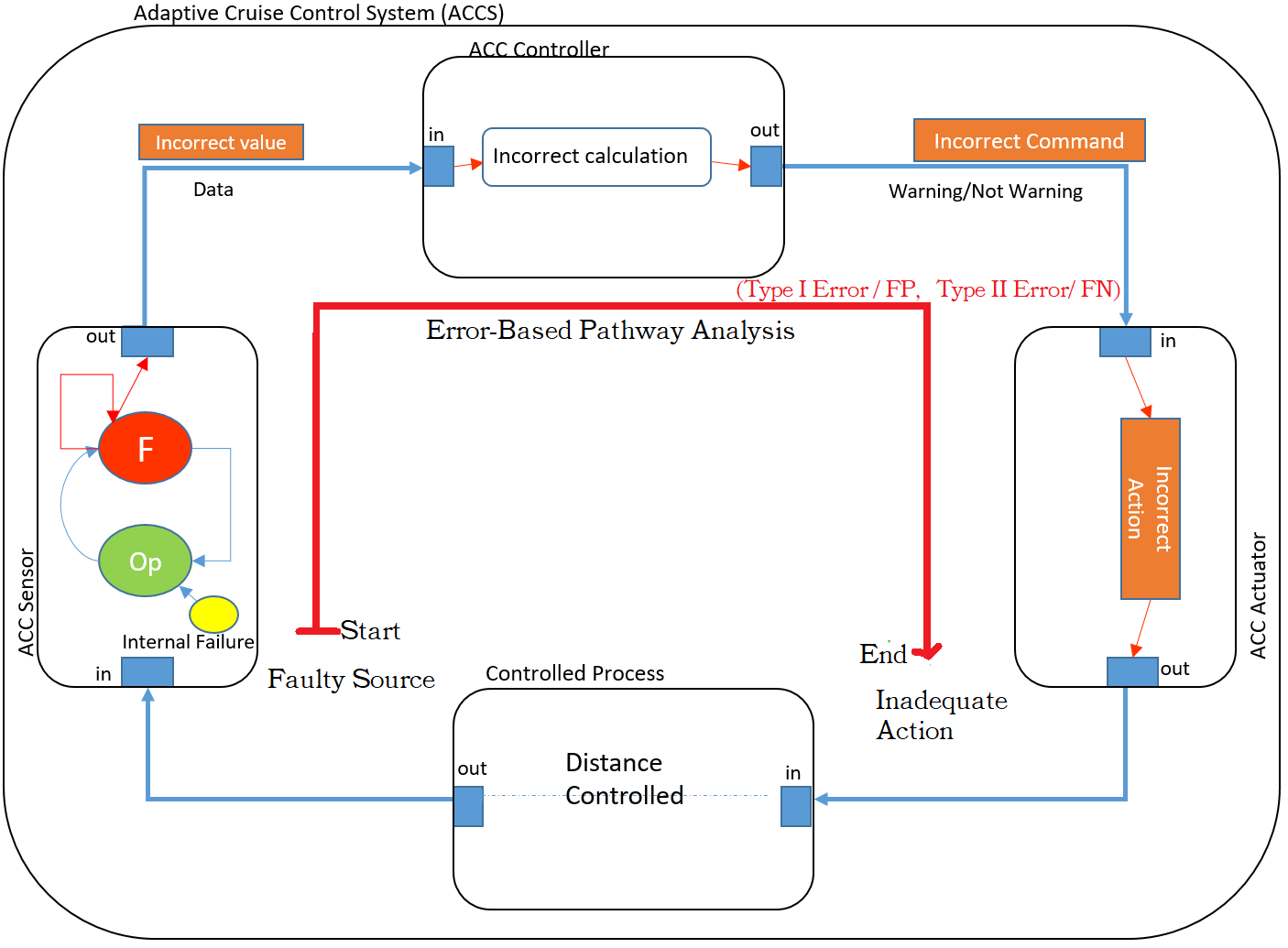

Supplement: S1 Fig — (PNG) [file pone.0299633.s001.png]
